# Postnatal skeletal growth is driven by the epiphyseal stem cell niche: potential implications to pediatrics

**DOI:** 10.1038/s41390-019-0722-z

**Published:** 2019-12-12

**Authors:** Andrei S. Chagin, Phillip T. Newton

**Affiliations:** 1 Department of Physiology and Pharmacology, Karolinska Institutet, 17177 Stockholm, Sweden; 2 Institute for Regenerative Medicine, Sechenov First Moscow State Medical University (Sechenov University), Moscow, Russian Federation; 3 Department of Women’s and Children’s Health, Karolinska Institutet and Pediatric Endocrinology Unit, Karolinska University Hospital, 17176 Stockholm, Sweden

## Abstract

Children’s longitudinal growth is facilitated by the activity of the growth plates, cartilage discs located near the ends of the long-bones. In order to elongate these bones, growth plates must continuously generate chondrocytes. Two recent studies have demonstrated that there are stem cells and a stem cell niche in the growth plate, which govern the generation of chondrocytes during the postnatal growth period. The niche, which allows stem cells to renew, appears at the same time as the secondary ossification center (SOC) matures into a bone epiphysis. Thus, the mechanism of chondrocyte generation differs substantially between neonatal and postnatal age, i.e., before and after the formation of the mineralized epiphyses. Hence, at the neonatal age bone growth is based on a consumption of chondro-progenitors whereas postnatally it is based on the activity of the stem cell niche. Here we discuss potential implications of these observations in relation to longitudinal growth, including the effects of estrogens, nutrition and growth hormone.

## Skeletal growth and the organization of epiphyseal cartilage

**Fig. 1:**
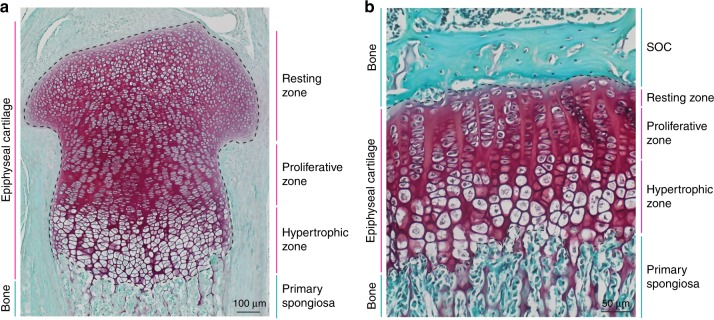
Development of the growth plate. Histological images of mouse epiphyseal cartilage before (**a**) and after (**b**) the growth plate is defined by the maturation of the secondary ossification center. Tissue sections from 3 days old (**a**) and 30 days old (**b**) mouse proximal tibiae are stained with Safranin O (red, cartilage) and Fast Green (green, bone and connective tissue).

**Fig. 2:**
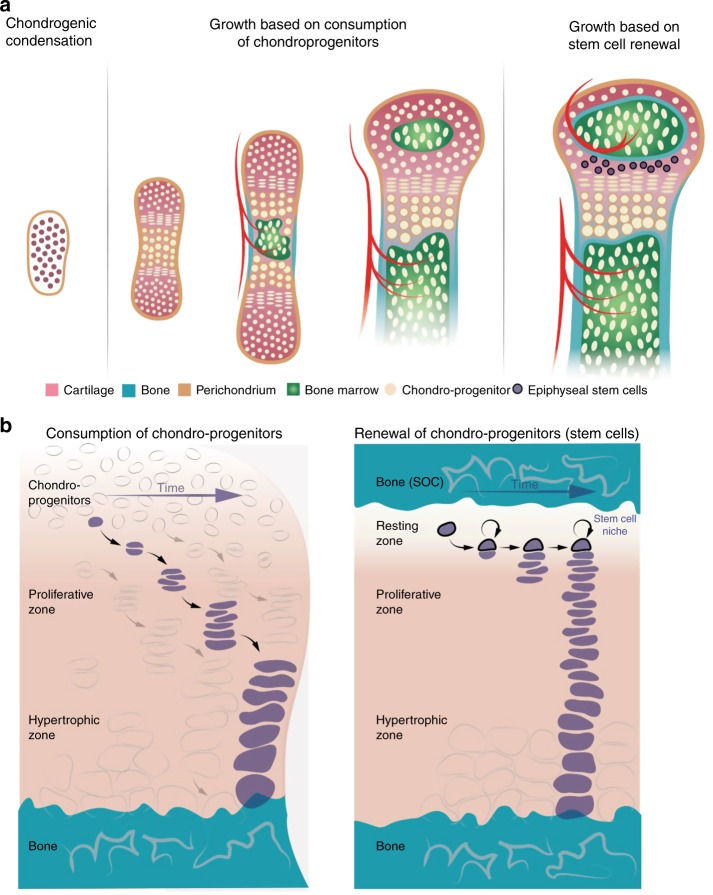
Two mechanisms of generation of epiphyseal chondrocytes. Schematic representation of different stages of bone growth (**a**) and mechanisms of chondrocyte generation before and after formation of the epiphyseal stem cell niche (**b**).

Children increase in height because of the activity of cartilage tissue located in the epiphyses (i.e., the proximal and distal ends) of the long-bones. During development, the epiphyses are comprised entirely of cartilage (Fig. 1a) and are separated from each other by bone tissue within the primary spongiosa. Shortly after birth a large proportion of cartilage cells (chondrocytes) in the center of each epiphysis are consumed to form the secondary ossification center (SOC). The SOC develops into a mature bone tissue (forming the bony epiphysis), which only leaves a narrow disc of epiphyseal cartilage between the two bony structures (i.e. the SOC and the primary spongiosa); these discs are called the (epiphyseal) growth plates^1^ and remain in the long-bones for the rest of the postnatal growth period (Fig. 1b, see scheme in Fig. 2a). This allocation of the epiphyseal cartilage into a spatially separated growth plate structure by the SOC is needed to protect growth plate chondrocytes from mechanical demands associated with weight bearing during juvenile growth.^2^ Importantly, the epiphyseal cartilage continuously facilitates bone growth before, during and after growth plate formation.

During all stages of long-bone growth, the epiphyseal cartilage can be divided histologically into three distinct zones: the resting, proliferative and hypertrophic zones (Fig. 1). The resting zone contains round chondrocytes in the least differentiated stage (chondro-progenitors), which divide rarely and whose progeny contribute to the proliferative zone.^3–5^ In the proliferative zone, chondrocytes divide rapidly and flatten, arranging themselves into columns that align parallel to the direction of growth. Thereafter, chondrocytes exit the cell cycle and further differentiate into large hypertrophic chondrocytes, forming the hypertrophic zone. Simultaneously, the cells remodel and calcify the cartilage surrounding them. Subsequently, the hypertrophic chondrocytes undergo apoptosis or trans-differentiation into osteoblasts,^6–8^ which leaves empty lacunae surrounded by calcified cartilage. The lacunae are invaded by blood vessels accompanied by osteo-progenitors, which use the calcified cartilage matrix as a scaffold on which to produce bone matrix. Hence, bone elongation is directly related to the size of the hypertrophic chondrocytes.^9^ The entire process is called endochondral bone formation and holds true for all long-bones in the body.

Since the process of long-bone growth requires an enormous number of hypertrophic chondrocytes, there is a continuous generation of hypertrophic chondrocytes in all growing children.

## New observations reveal a novel mechanism of chondrocyte generation

Two recent studies shed light on mechanisms how an abundant and continuous production of chondrocytes is achieved once the epiphyseal cartilage is reduced to narrow growth plates in the postnatal period.^10,11^ Studying cell kinetics within the growth plates of mice revealed that in fetal and neonatal life, bones grow by the recruitment and gradual consumption of chondro-progenitors.^11^ However, as soon as SOCs develop, the mechanism of bone growth changes dramatically and instead of being consumed, chondro-progenitors start to renew themselves, thereby not only generating chondrocytes in the underlying zones but also maintaining their own population.^11^ Furthermore, the formation of the SOC above the resting zone causes the formation of a stem cell niche in the growth plate, which we named the “epiphyseal stem cell niche”.^11^ Upon formation of this niche, chondro-progenitors acquire markers of stem cells and substantially change their gene expression profile.^11^ Almost at the same time, Mizuhashi et al. showed that postnatal growth is driven by a population of PTHrP-positive chondro-progenitors, which are located in the resting zone and can facilitate the generation of chondrocytes without being consumed.^10^ These PTHrP-positive chondro-progenitors are named by the authors “unique skeletal stem cells”,^10^ which can generate chondrocytes that subsequently transdifferentiate into osteoblasts^6,7^ and stromal cells,^10^ thereby contributing to different cellular populations in the skeleton. Herein, we propose the name “epiphyseal stem cells” located in the corresponding epiphyseal niche formed in the epiphysis by the SOC,^11^ to distinguish this population from other types of skeletal stem cells recently reported.^8,12–16^

Taken together, these studies show that there is a conceptual difference between neonatal and postnatal bone growth, the former is based on the consumption of chondro-progenitors whereas the latter is based on stem cells and a corresponding stem cell niche, which facilitates their maintenance (Fig. 2a, b). There are several implications to this discovery.

## The concept of the epiphyseal stem cell niche

The concept of the stem cell niche as a specific microenvironment that promotes the renewal of progenitor cells was put forth by R. Schofield in 1978.^17^ These microenvironments are highly variable for different stem cell niches and can influence stem cell behavior in many different ways via interactions with neighboring cell types, intricate networks and gradients of signaling molecules, extracellular matrix components, and mechanical forces. For example, adult hematopoietic stem cells (HSCs) require interaction with endothelial cells and CXC-chemokine ligand 12 (CXCCL12)-abundant reticular (CAR) mesenchymal stromal cells for their renewal, both highly expressing CXCCL12 and stem cell factor,^18–20^ inter-follicular epidermis (IFE) stem cells require attachment to the basement membrane,^21^ and mesenchymal stem cells are supported by mechanical stimuli.^22^ Even composition of one niche may be very complex and include a variety of cell types and environmental factors, as for the HSCs niche, on top of CAR and endothelial cells, it includes macrophages, osteo-progenitors, low oxygen tension, non-myelinating Schwann cells, and sympathetic innervation (for review see refs. ^19,20^).

In the case of the growth plate, the niche microenvironment arises simultaneously with the formation of the bony epiphysis (i.e., once the SOC has matured) directly above the layer of chondro-progenitors (Fig. 1b). Thus, the interaction between bone tissue and epiphyseal stem cells can cause their renewal either via direct interaction, changes in extracellular matrix (ECM) or diffused morphogens.^11^

The formation of various stem cell niches occurs late in development or even postnatally. For example, in mice hematopoietic stem cells home to bone on embryonic day E17.5,^23^ renewal of hair follicles appears with the start of the hair cycle around postnatal day 17,^24^ the germline stem cell niche begins to function properly upon sexual maturation^25^ and renewal of articular chondro-progenitors begins after joint cavitation.^26,27^ The formation of the epiphyseal stem cell niche upon the postnatal development of bony epiphyses fits into this pattern well.

Generally, tissue functionality on the basis of a stem cell niche can be divided into three steps. First, stem cells divide slowly generating either new stem cells or more-committed immediate progeny via either invariant or populational asymmetry.^28^ Second, the more-committed progeny cells proliferate extensively, forming a pool of so-called transit-amplifying cells (TACs). Third, TACs differentiate into nonproliferative cells harboring tissue-specific function(s).^21,29^ Cell kinetics in the growth plate perfectly reflect this functionality. Indeed, slow-dividing resting zone cells correspond to stem cells, their fast proliferating progeny of the proliferative zone correspond to TACs and hypertrophic chondrocytes correspond to terminally differentiated cells that harbor the functional role of bone elongation.^10,11^ It is tempting to consider the IFE of the skin and the growth plate to be analogous in terms of functional organization. In both cases continuous production of terminally differentiated cells of a single type (keratinocytes of stratified squamous epithelium and hypertrophic chondrocytes, respectively) is required. In the IFE, slowly dividing stem cells in the basal layer generate TACs, which thereafter differentiate further. Similarly, in the growth plate slowly dividing chondro-progenitors generate highly proliferative flat chondrocytes (i.e., TACs), which thereafter terminally differentiate into the hypertrophic chondrocytes required for bone elongation. It is fascinating that the presence of stem cells in both organs was proposed as early as the 1970s, on the basis of heterogeneity in the length of the cell cycle, as revealed by the then-novel procedure of ^3^H-thymidine labeling.^30,31^

## Fusion of the growth plate and the stem cell niche

One potential mechanism influencing the epiphyseal stem cell niche is the expression of the morphogen Sonic hedgehog (Shh) by cells located in the bony epiphyses.^11^ Hedgehog signaling is well-known to be involved in the maintenance of stem cell niches.^32^ Pharmacological activation of hedgehog signaling promotes proliferation of the cells in the niche,^11^ but if administered prior to the formation of the niche, it inhibits column formation.^10^ Inhibition of the hedgehog signaling abrogates division of stem cells and causes fusion of the growth plate.^10,11^ Thus, it is plausible to hypothesize that fusion of the growth plate can be caused by disruption of the epiphyseal stem cell niche. High levels of estrogens are a well-known cause of growth plate fusion;^33^ if this effect is mediated via direct modulation of hedgehog signaling or the general composition of the niche is yet to be determined. Interestingly, in the absence of estrogen or estrogen receptor alpha, humans do not fuse their growth plates and growth is virtually unlimited.^34,35^ Thus, theoretically, cessation of growth might not be caused by the entire consumption of chondro-progenitors, but by modulation of the stem cell niche so that it no longer facilitates stem cell renewal, leading to fusion of the growth plate and the cessation of growth. Such a relationship between the niche and fusion of the growth plate, appears likely, but remains to be proven. The fact that fusion of the growth plate is very abrupt and does not involve cell death^36,37^ aligns with the concept that without continuous generation of chondrocytes, the growth plate will be eroded rapidly by the underlying bone.

## Growth hormone, nutrition and growth

Growth hormone (GH) is a key stimulant of children’s growth and is commonly used to treat children with retarded growth. However, some actions of GH remain to be explained, such as the relatively small increase in final height, and an unresponsiveness to the therapy in some children.^38^ Some of the effects of GH are mediated via insulin-like growth factor I (IGF1), but direct action on the growth plate has also been shown,^39^ which has been proposed to be specifically on the resting zone cells.^40^ Interestingly, the growth-promoting action of GH appears between 2 and 3 weeks of age in mice,^41^ coinciding with the appearance of epiphyseal stem cells and the niche.^10,11^ Combining all these indirect observations, it is plausible to hypothesize that GH can act specifically on epiphyseal stem cells or to modulate their niche. The action of GH on the epiphyseal stem cells and composition of the niche remains to be elucidated.

mTOR complex 1 (mTORC1) is a nutritional sensor^42^ and accelerates the differentiation of intestinal stem cells and neural stem cells,^43,44^ whereas it promotes the initial expansion of hematopoietic stem cells.^45^ In the epiphyseal stem cell niche, activation of mTORC1 promotes the symmetrical division of stem cells, thereby increasing their number,^11^ likely in an autophagy-independent manner.^46^ In genetic experiments the activation of mTORC1 does not lead to bone elongation, perhaps due to the gradual disorganization of the growth plate.^47^ However, temporal activation of mTORC1, such as with high levels of amino acids or IGF1 (both major activators of mTORC1), may, in theory, increase the number of the epiphyseal stem cells.

## Growth patterns and evolution

The basic mechanism of endochondral ossification is about 400 million years old and has been described for lobe-finned and bony fish as well as for all terrestrial vertebrates. However, not all of these vertebrates form SOCs in their epiphyses and therefore live their whole lives with entirely cartilaginous epiphyses (e.g. crocodilians, urodela, teleost). Interestingly, these animals continue to grow throughout their lifetimes, a feature called indeterminate growth, which is believed to be made possible by the presence of a virtually unlimited number of chondro-progenitors that constitute the entire epiphysis. The appearance of the SOC and allocation of the growth plate into a spatially separated organ seems to be an adaptation to the life on land.^2^ However, SOCs are formed at the expense of chondro-progenitors, theoretically limiting the growth potential of all terrestrial vertebrates. Despite this, certain mammals undergo indeterminate growth despite possessing a relatively small number of chondro-progenitors (e.g., rodents, elephants). Indeterminate growth also occurs in humans in the absence of estrogen or estrogen receptor alpha.^34,35^ Thus, the formation of an epiphyseal stem cell niche may overcome constraints associated with evolution of bony epiphyses^2^ and the associated loss of chondro-progenitors. The evolutionary advantages and disadvantages of determinate vs. indeterminate growth remain elusive, but the appearance of the stem cell niche could, in theory, be the key to explaining how taxa with SOCs, including humans as well as all other mammals, can have such flexible growth strategies.

In conclusion, the discovery of the epiphyseal stem cell niche and epiphyseal stem cells, which are responsible for longitudinal growth postnatally, provides a novel perspective on growth regulation per se as well as providing an alternative view of currently unexplained clinical observations. However, a direct extrapolation of observations made from mice to human physiology should be made with caution, because their mechanisms of growth are not identical.
